# Erythropoiesis and Blood Pressure Are Regulated via AT1 Receptor by Distinctive Pathways

**DOI:** 10.1371/journal.pone.0129484

**Published:** 2015-06-24

**Authors:** Hideki Kato, Junji Ishida, Taiji Matsusaka, Tomohiro Ishimaru, Keiji Tanimoto, Fumihiro Sugiyama, Ken-ichi Yagami, Masaomi Nangaku, Akiyoshi Fukamizu

**Affiliations:** 1 Life Science Center, Tsukuba Advanced Research Alliance (TARA), University of Tsukuba, Tsukuba, Ibaraki, 305–8577, Japan; 2 Graduate School of Life and Environmental Sciences, University of Tsukuba, Tsukuba, Ibaraki, 305–8577, Japan; 3 Laboratory Animal Resource Center, University of Tsukuba, Tsukuba, Ibaraki, 305–8577, Japan; 4 Division of Nephrology and Endocrinology, The University of Tokyo Graduate School of Medicine, Hongo, Bunkyo, Tokyo, 113–8655, Japan; 5 Institute of Medical Sciences, Tokai University School of Medicine, Bohseidai, Isehara, Kanagawa, 259–1193, Japan; Max-Delbrück Center for Molecular Medicine (MDC), GERMANY

## Abstract

The renin–angiotensin system (RAS) plays a central role in blood pressure regulation. Although clinical and experimental studies have suggested that inhibition of RAS is associated with progression of anemia, little evidence is available to support this claim. Here we report that knockout mice that lack angiotensin II, including angiotensinogen and renin knockout mice, exhibit anemia. The anemia of angiotensinogen knockout mice was rescued by angiotensin II infusion, and rescue was completely blocked by simultaneous administration of AT1 receptor blocker. To genetically determine the responsible receptor subtype, we examined AT1a, AT1b, and AT2 knockout mice, but did not observe anemia in any of them. To investigate whether pharmacological AT1 receptor inhibition recapitulates the anemic phenotype, we administered AT1 receptor antagonist in hypotensive AT1a receptor knockout mice to inhibit the remaining AT1b receptor. In these animals, hematocrit levels barely decreased, but blood pressure further decreased to the level observed in angiotensinogen knockout mice. We then generated AT1a and AT1b double-knockout mice to completely ablate the AT1 receptors; the mice finally exhibited the anemic phenotype. These results provide clear evidence that although erythropoiesis and blood pressure are negatively controlled through the AT1 receptor inhibition *in vivo*, the pathways involved are complex and distinct, because erythropoiesis is more resistant to AT1 receptor inhibition than blood pressure control.

## Introduction

The renin–angiotensin system (RAS) plays an important role in blood pressure regulation and fluid homeostasis in the circulatory system. In addition, it exerts various actions in diverse target tissues. Angiotensin II (Ang II), a major determinant of blood pressure, is produced from a precursor substrate, angiotensinogen (Agt), via an enzymatic cascade of two proteases, renin (Ren) and angiotensin converting enzyme (ACE). Diverse actions of Ang II are mediated by several Ang II receptor subtypes present in a variety of target tissues. Two Ang II receptor subtypes, designated as Ang II type 1 (AT1) and Ang II type 2 (AT2), have been identified in humans. In rodents, two AT1 receptor isoforms, AT1a and AT1b, have been identified, and blood pressure regulation is mediated mainly through the AT1a receptor, which is expressed at higher levels than the AT1b receptor in most organs [1]. No apparent phenotype has been identified in AT1b receptor knockout mice (AT1b(-/-)) [2].

Although a role for RAS in the regulation of erythropoiesis has long been suspected, its mechanistic basis is complex and remains incompletely understood. Using genetically engineered mice and bone marrow transplantation, we previously showed *in vivo* that activation of RAS led to enhanced erythropoiesis, mainly through the elevation of plasma erythropoietin (Epo) levels via kidney AT1a receptor [3, 4]. Our results have also shown that genetic ablation of AT1a receptor in the presence of activated RAS condition restored erythrocytosis to normal levels [4], although AT1a receptor knockout mice [AT1a(-/-)] themselves had no apparent erythropoietic phenotype. Furthermore, constitutively active AT1a receptor knock-in mice have significantly elevated hemoglobin levels [5]. These results indicate that RAS activation induces erythropoiesis via the AT1 receptor *in vivo*.

An association between RAS inhibition and impaired erythropoiesis has also been reported in numerous clinical cases in which ACE inhibitors (ACEi) were used [6–18]. AT1 receptor blockers (ARBs) have been reported to decrease the erythrocyte number in many clinical conditions [16, 19–23]. This issue remains controversial, however, because many other clinical studies revealed no inhibitory effects of ACEi or ARB on erythropoiesis [24–31]. In addition, the possible role of AT2 receptor in erythropoiesis has not been investigated. A lack of definite *in vivo* evidence on this issue has contributed to confusion in the clinic [32].

In this study, we investigated the correlation between the status of RAS inhibition and erythropoiesis using genetically engineered mice in combination with pharmacological challenges.

## Materials and Methods

### Animal model

Agt knockout mice [Agt(-/-)], Ren knockout mice [Ren(-/-)], AT1a(-/-), and mice that were heterozygous for AT1a receptor-null and homozygous for AT1b receptor-null [AT1a(+/-) AT1b(-/-)] were generated in our laboratory as previously described [33–36]. Double-homozygous AT1a(-/-) AT1b(-/-) mice were generated by cross-mating AT1a(+/-) AT1b(-/-) mice, and genotypes were determined by Southern blot analysis as previously described [36]. AT2 receptor knockout mice [AT2(-/-)] were kindly provided by Dr. T. Inagami (Vanderbilt University) [37]. All mice were of the C57/BL6J background. All mice were fed standard mouse chow and water ad libitum. Animal experiments were carried out during anesthesia with pentobarbital sodium and in a humane manner, under the approval of the Institutional Animal Experiment Committee of the University of Tsukuba (Permit Number: 07–118) and the University of Tokai (Permit Number: 104011), and in accordance with the Regulations for Animal Experiments of our university and the Fundamental Guidelines for Proper Conduct of Animal Experiment and Related Activities in Academic Research Institutions under the jurisdiction of the Ministry of Education, Culture, Sports, Science, and Technology.

### Measurement of hematocrit, reticulocyte counts, and plasma erythropoietin levels

Three-month-old male mice were anesthetized with pentobarbital sodium, and 200 μL of blood was collected via the tail vein into a tube containing EDTA. Hematopoietic indices were determined using an automatic counter (Nihon Koden, Tokyo, Japan). The number of reticulocytes was determined by staining peripheral blood with Diff-Quick (Sysmex, Kobe, Japan); reticulum-positive cells per 1,000 red blood cells were counted for sample under a microscope. Plasma was also centrifuged for 10 min at 3,000 *g* and stored at -80°C until use, and Epo concentrations were analyzed using a commercial ELISA kit (Roche Diagnostics, Germany).

### Bloodletting experiment

Bloodletting was performed in C57BL/6J wild-type (WT) and Agt(-/-) mice. In preliminary experiments, 0.3 mL of bloodletting from tail vein of WT decreased hematocrit levels close to those of Agt(-/-). Therefore, WT and Agt(-/-) mice were anesthetized, and 0.3 mL of peripheral blood was extracted. After 24 h, hematopoietic indices and plasma Epo levels were measured as described above.

### Measurement of blood pressure

Heart rate and systolic, mean, and diastolic blood pressure were measured using a non-invasive computerized tail-cuff blood pressure system for mice (BP-98A; Softron, Japan) as described previously [38].

### Ang II infusion and ARB administration into angiotensinogen knockout mice

Basal blood pressure level of Agt(-/-) was measured before drug administration. The mice were then anesthetized with pentobarbital sodium, and 100 μl of blood was collected via the tail vein and hematopoietic indices were determined as described above. The mice were weighed, and an Alzet model 2004 mini-osmotic pump (DURECT Corp. California. USA) was implanted subcutaneously into the dorsum of each mouse. Agt(-/-) were assigned to three groups (n = 5 /group). First group received a mini-pump delivering Ang II (Peptide Institute, Osaka, Japan) at a final dose of 0.3 mg/kg/day diluted in sterile 0.9% saline for 4 weeks. The second group received a mini-pump delivering Ang II at the same dose, and also received losartan (30 mg/kg/day), an ARB in the drinking water. This regimen of losartan treatment inhibits pressor responses of exogenous infusion of Ang II and further lowers blood pressure of AT1a(-/-) to the levels of Agt(-/-) or AT1a(-/-) AT1b(-/-) [36, 39]. The third group received a mini-pump delivering saline, as a control. Blood pressures and hematopoietic indices were measured as described above.

### Administration of an ARB, losartan, in AT1a receptor knockout mice

Blood pressure and hematopoietic indices of 3-month-old male AT1a(-/-) mice were determined as described above. These animals were then treated with 30 mg/kg/day of losartan in the drinking water for 8 weeks. Blood pressures and hematopoietic indices were measured.

### Statistical analysis

Data are presented as means ± SD. Statistical significance between groups was evaluated by unpaired Student’s *t* test. A *p* value < 0.05 was considered statistically significant. We used one-way ANOVA to test for differences among more than three groups. Additionally, Tukey's HSD tests were performed post-hoc for specific comparisons between groups (JMP Pro Version 11.0.0, SAS Institute Inc., Cary, NC, USA).

## Results

### Anemia associated with lack of Ang II production

**Fig 1:**
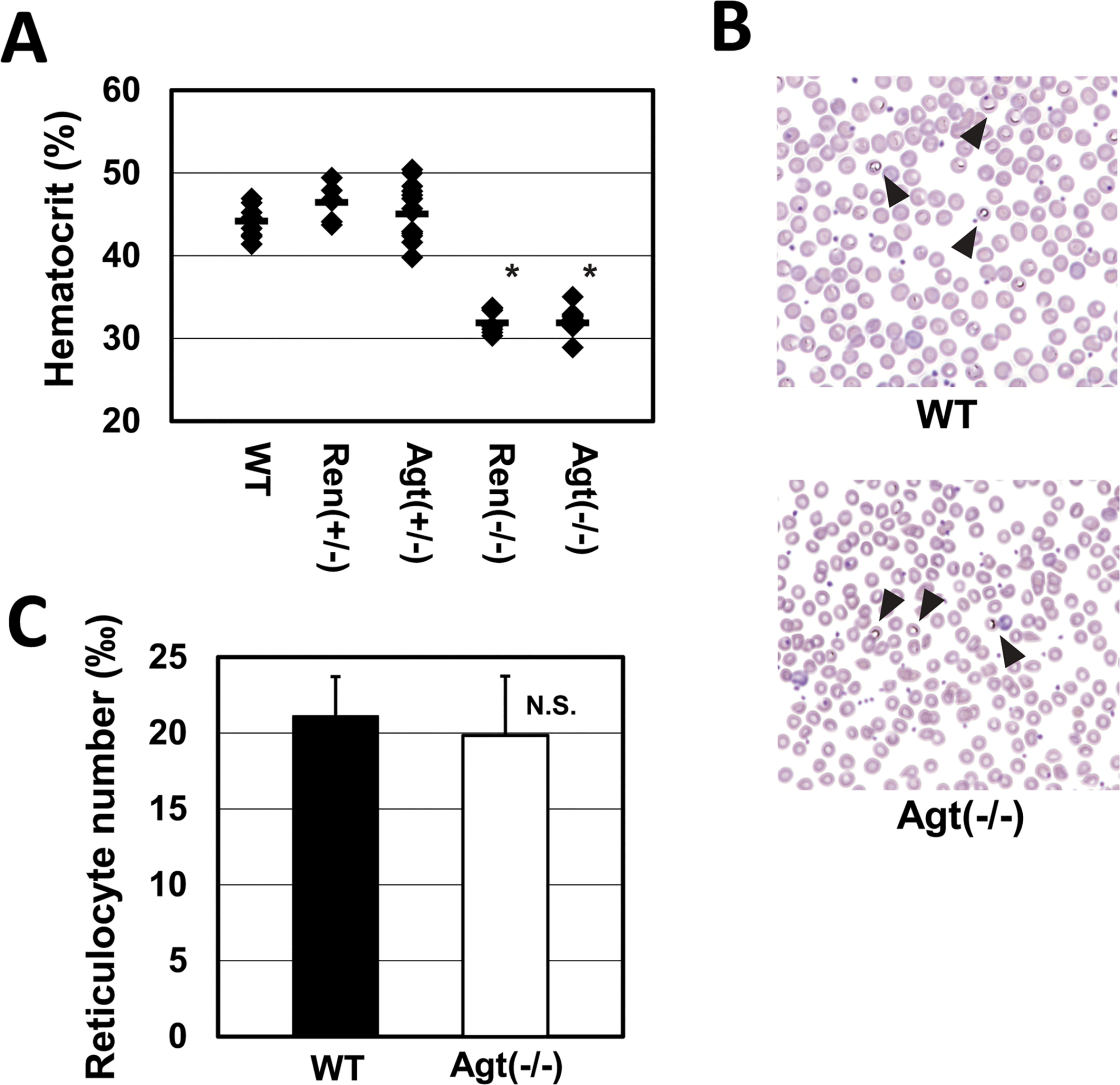
Hematopoietic characteristics of knockout mice lacking Ang II production. (A) Hematocrit was measured for WT (n = 10), Ren(+/-) (n = 6), Agt(+/-) (n = 15), Ren(-/-) (n = 9), and Agt(-/-) (n = 13) mice; the animals were 3 months old. Individual measurements are indicated by a (◆). The group mean is represented by a thick horizontal bar (*p < 0.0001 vs. WT, ANOVA followed by Tukey’s HSD post hoc comparisons). (B) Panels show smears of peripheral blood. Peripheral blood smears of Agt(-/-) appear normal. Arrows indicate methylene blue–stained reticulocytes. (C) Mean reticulocyte counts of WT (n = 10) and Agt(-/-) (n = 13) are represented. Values shown are means ± SD. N.S., not significant.

Targeted disruption of angiotensinogen or renin gene caused anemia in 3-month-old male mice with a 30% reduced hematocrit level relative to the WT [Agt(-/-), 31.87 ± 1.61% (n = 13) and Ren(-/-), 31.86 ± 1.33% (n = 9); (both *p* < 0.0001, ANOVA followed by Tukey’s HSD post hoc comparisons) vs. WT, 44.46 ± 1.68% (n = 10)] (Fig 1A). Consistent with that, circulating hemoglobin levels were also significantly reduced in both Agt(-/-) and Ren(-/-) (Table 1). Evaluation of peripheral blood from the heterozygous deficient mice, *i*.*e*., Agt(+/-) and Ren(+/-), revealed normal hematocrit values [Agt(+/-), 45.03 ± 3.33% (n = 15) and Ren(+/-), 46.42 ± 2.19% (n = 6)]. Considering the almost identical degree of severity of anemia in Agt(-/-), Ren(-/-) (in this study) and ACE knockout mice (previously reported in [40]), we speculate that the anemic phenotype observed in these animals is attributable to complete loss of Ang II production, rather than the accumulation of substrates of or the loss of products (other than Ang II) of ACE or renin enzymes, because Ang II and its fragments, such as Ang (1–7) or Ang III, are the only common peptides produced from angiotensinogen, renin, and ACE. Gross abnormalities in platelets or white cell numbers were not found in Agt(-/-) nor Ren(-/-) (Table 1). Furthermore, no significant differences in the mean corpuscular volume (MCV), mean corpuscular hemoglobin (MCH), and mean corpuscular hemoglobin concentration (MCHC) were found between the groups, indicating that Agt(-/-) and Ren(-/-) are normocytic and normochromic anemia (Table 1). We decided to use Agt(-/-) in the following experiments, because angiotensinogen is a unique substrate in the renin–angiotensin system; therefore, possible effects exerted by unknown substrates of renin or ACE on erythropoiesis can be ruled out.

**Table 1:** Peripheral blood count of each mouse.

	WT	Agt(-/-)	Ren(-/-)	AT1a(-/-)	AT2(-/-)	AT1a(-/-)
						AT1b(-/-)
**Hct**	**44.46 ± 0.78**	**31.87 ± 0.53** *	**31.86 ± 0.44** *	**42.88 ± 0.52**	**47.38 ± 0.68**	**33.18 ± 1.35** *
**RBC**	**910 ± 19.7**	**667 ± 11.2** *	**661 ± 14.7** *	**913 ± 16.6**	**966 ± 11.4**	**686 ± 29.95** *
**Hemoglobin**	**15.05 ± 0.28**	**10.75 ± 0.17** *	**10.54 ± 0.22** *	**13.96 ± 0.27**	**15.97 ± 0.20**	**11.8 ± 0.52** *
**MCV**	**48.9 ± 0.28**	**47.85 ± 0.16**	**48.22 ± 0.52**	**46.278 ± 0.37**	**49.0 ± 0.23**	**48.3 ± 0.88**
**MCH**	**16.53 ± 0.089**	**16.13 ± 0.149**	**15.95 ± 0.100**	**15.07 ± 0.126**	**16.51 ± 0.082**	**16.7 ± 0.23**
**MCHC**	**33.86 ± 0.18**	**33.78 ± 0.27**	**33.07 ± 0.33**	**32.58 ± 0.38**	**33.72 ± 0.19**	**34.64 ± 0.25**

* *p* < 0.0001 vs. WT.

Anemia can be ascribed to two major etiologies: the destruction of red blood cells or reduced erythrocyte production. To reveal the cause of anemia, we observed the morphology of peripheral blood smears from Agt(-/-) mice, and found them to be normal (Fig 1B). We then counted the reticulocyte number in peripheral blood of Agt(-/-) mice and found it to be slightly reduced but not significantly different from the WT (WT, 21.1 ± 2.60x (n = 10) vs. Agt(-/-), 19.8 ± 3.90‰ (n = 13); *p* = 0.426) (Fig 1C).

### Correlation between the absence of Ang II and plasma erythropoietin levels

**Fig 2:**
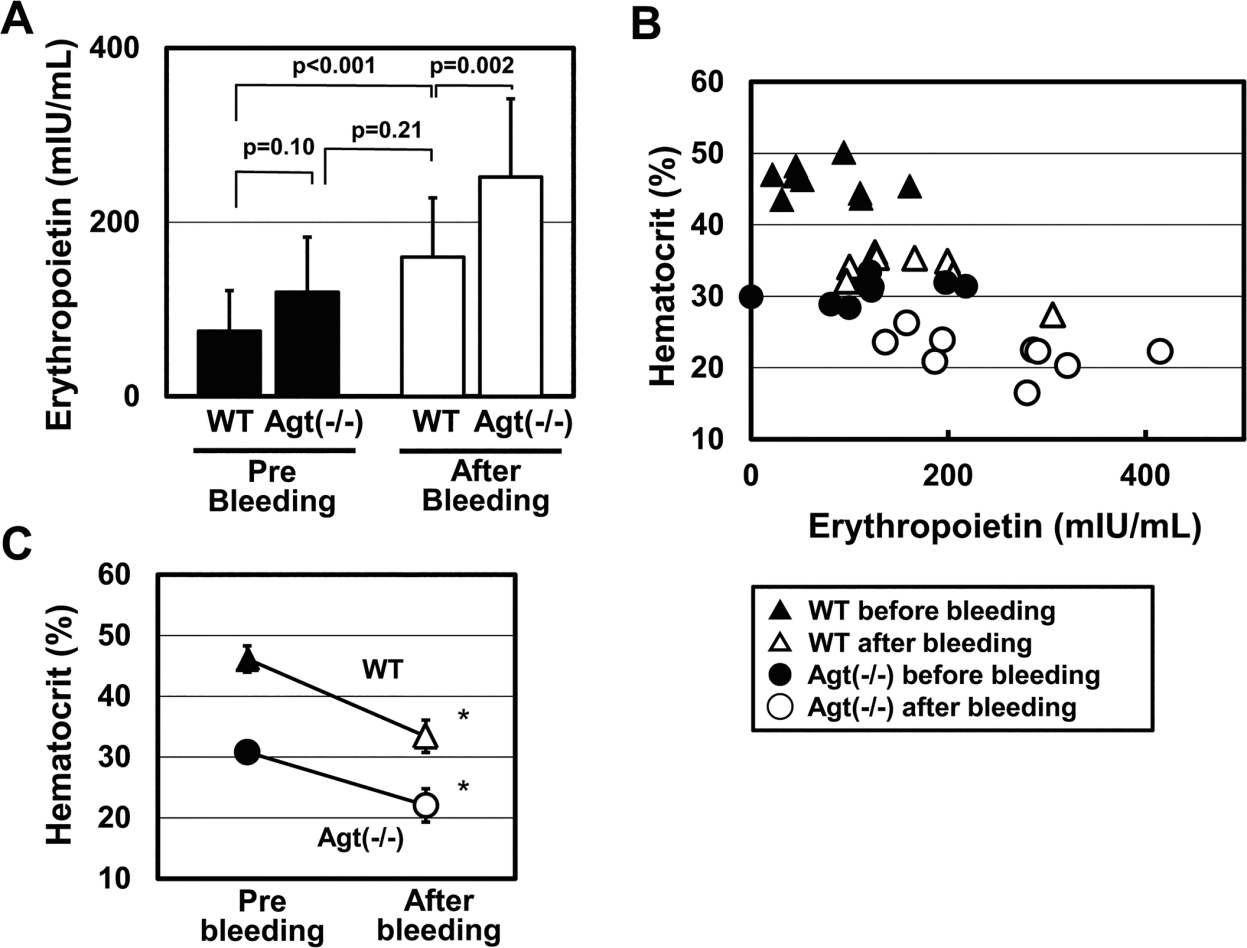
Plasma erythropoietin levels of WT and Agt(-/-). (A) Plasma Epo was measured for WT and Agt(-/-), and the group mean is represented. One day after 0.3 mL of bleeding, plasma Epo levels and hematocrit levels were measured (n = 9 for each group). (B) Relationship between hematocrit and plasma Epo levels before and after bleeding. The individual mouse data are represented by marks. (C) Hematocrit values of WT and Agt(-/-) before and after 0.3 mL of bloodletting. *p < 0.001 vs. pre-bleeding.

Epo is the major hormonal regulator of erythropoiesis. In humans, the relationship between the use of drugs, such as ACEi or ARB, and plasma Epo levels remains controversial [32]. We thought this issue could be assessed *in vivo* by examining plasma Epo levels in Agt(-/-). As shown in Fig 2A and 2B, plasma Epo levels in Agt(-/-) were slightly higher, but not statistically different from those in WT (WT, 74.78 ± 46.69 mIU/mL (n = 9); Agt(-/-), 119.80 ± 63.15 mIU/mL (n = 9); *p* = 0.10).

To determine whether slightly elevated plasma Epo levels in Agt(-/-) were within the range of physiologic response to anemia, we compared the Epo levels in Agt(-/-) to those in WT mice in which anemia had been artificially induced to an equivalent severity. To this end, WT mice were bled (0.3 mL from tail vein), and complete blood counts and plasma Epo levels were measured before and 24 h after the procedure. As shown in Fig 2C, 0.3 mL of bleeding induced anemia in WT [33.41 ± 2.66% (n = 9)] to a hematocrit level equivalent to that seen in Agt(-/-). Although plasma Epo levels in anemic WT significantly increased after bleeding (WT after bleeding: 160.01 ± 67.83 mIU/mL (n = 9), respectively; *p* < 0.001 vs. WT pre-bleeding), it was not significantly different from that of non-treated Agt(-/-) (*p* = 0.21) (Fig 2A and 2B).

In order to further elucidate the capability of the Agt(-/-) to increase Epo in response to anemic conditions, we subjected Agt(-/-) mice to the same bleeding procedure described above. As shown in Fig 2C, 0.3 mL of bleeding of Agt(-/-) mice caused more severe anemia, and hematocrit levels decreased to 22%. In addition, plasma Epo levels in Agt(-/-) mice significantly increased after bleeding [before treatment: 119.80 ± 63.15 mIU/mL (n = 9); after treatment, 251.83 ± 89.95 mIU/mL (n = 9); *p* = 0.002] (Fig 2A and 2B). These results suggest that the anemia of Agt(-/-) mice was not the result of reduced Epo levels, and that the Epo response in Agt(-/-) mice was not impaired.

### Recovery of anemia of angiotensinogen knockout mice by Ang II infusion

Anemia in ACE knockout mice can be recovered by infusion of a small dose of Ang II [40], which has not yet been tested in Agt(-/-) mice. Furthermore, even if Ang II infusion were able to rescue anemia in Agt(-/-) mice, it remains to be determined which receptor subtypes, AT1, AT2 receptors, or receptors for the sub-fragments of Ang II (*i*.*e*. Ang (1–7) and Ang III, etc.), would be responsible for the recovery. To investigate this, we treated Agt(-/-) mice with Ang II alone, or Ang II plus losartan.

**Fig 3:**
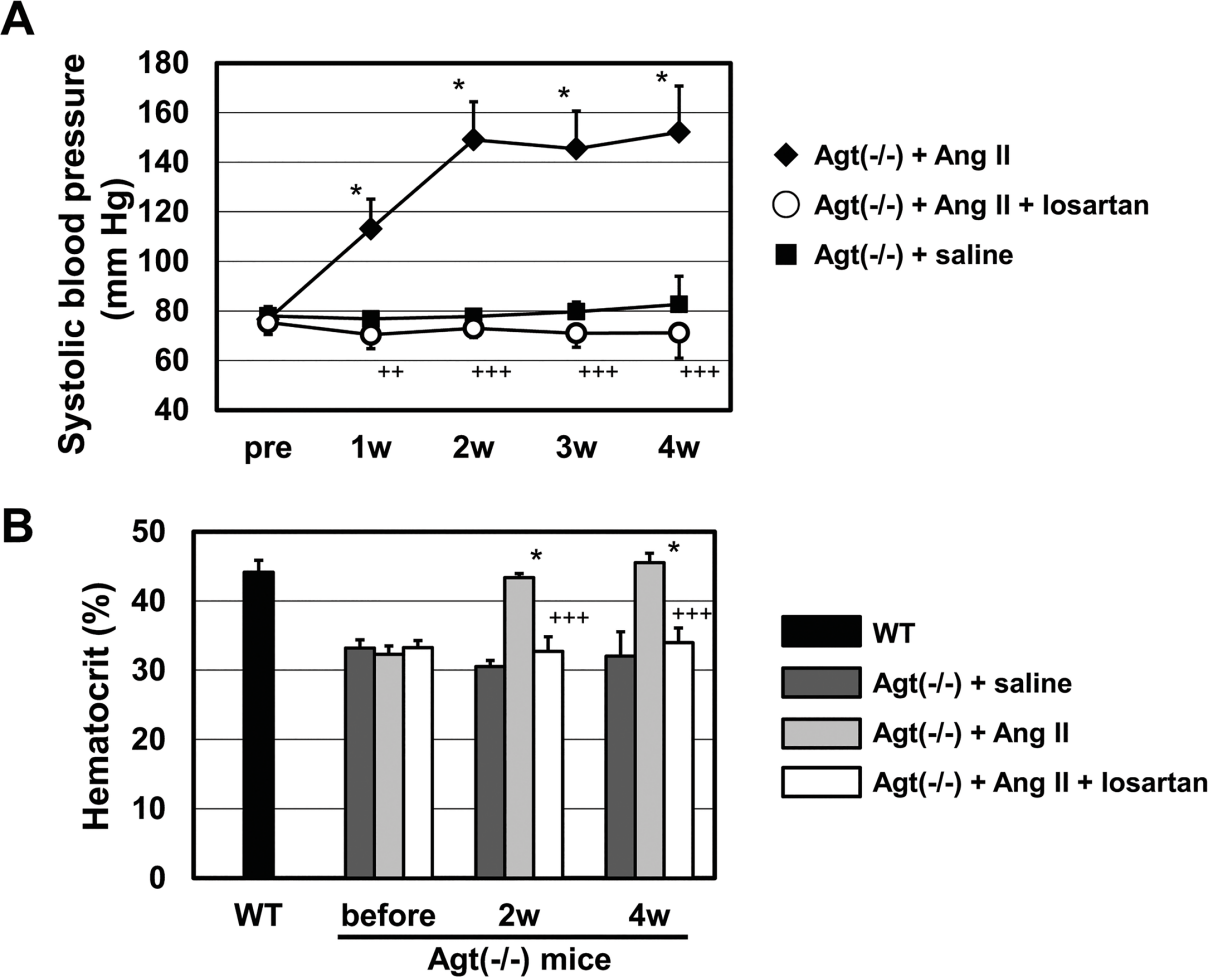
Systolic blood pressure and hematocrit values in Agt(-/-) mice after chronic administration of Ang II, with or without oral administration of losartan. (A) Changes in blood pressure in Agt(-/-) mice following subcutaneous chronic infusion of Ang II (0.3 mg/kg/day) (n = 4), Ang II + losartan (30 mg/kg/day) (n = 4), or saline (n = 5). (B) Changes in hematocrit values of Agt(-/-) by subcutaneous chronic infusion of Ang II (n = 4), Ang II + losartan (n = 4), and saline (n = 5). The hematocrit value of WT is shown for comparison. ***p < 0.001 vs. saline group. ^++^ *p* < 0.01, ^+++^ *p* < 0.001 vs. Ang II group in Fig 3A and 3B.

Agt(-/-) was infused with a subpressor dose of 0.3 mg/kg/day of Ang II for 4 weeks by osmotic mini-pump. This small dose of Ang II exerts minimal effects on blood pressure and hematocrit value in WT mice [40]. Systolic blood pressure and hematocrit levels of Agt(-/-) mice before and after treatment are shown in Fig 3A and 3B. Agt(-/-) mice were highly susceptible to Ang II infusion, and this subpressor dose of Ang II increased systolic blood pressure of Agt(-/-) mice to roughly 140 mmHg 1 week after infusion (Fig 3A). Also, Ang II infusion markedly recovered the hematocrit value in Agt(-/-) mice, bringing it close to that of the baseline WT levels 2 weeks after treatment; this level was sustained for up to 4 weeks (Fig 3B). Similar to the previous result obtained in ACE knockout mice [40], these results indicate that the absence of Ang II is associated with anemia as well as hypotension in Agt(-/-). Furthermore, recovery of both blood pressure and hematocrit levels were completely inhibited by simultaneous oral administration of ARB (Fig 3A and 3B). Thus, we conclude that both hematopoietic and blood pressure phenotypes in Agt(-/-) are mediated via Ang II-AT1 receptor pathways *in vivo*.

### Absence of anemia in Ang II receptor knockout mice

**Fig 4:**
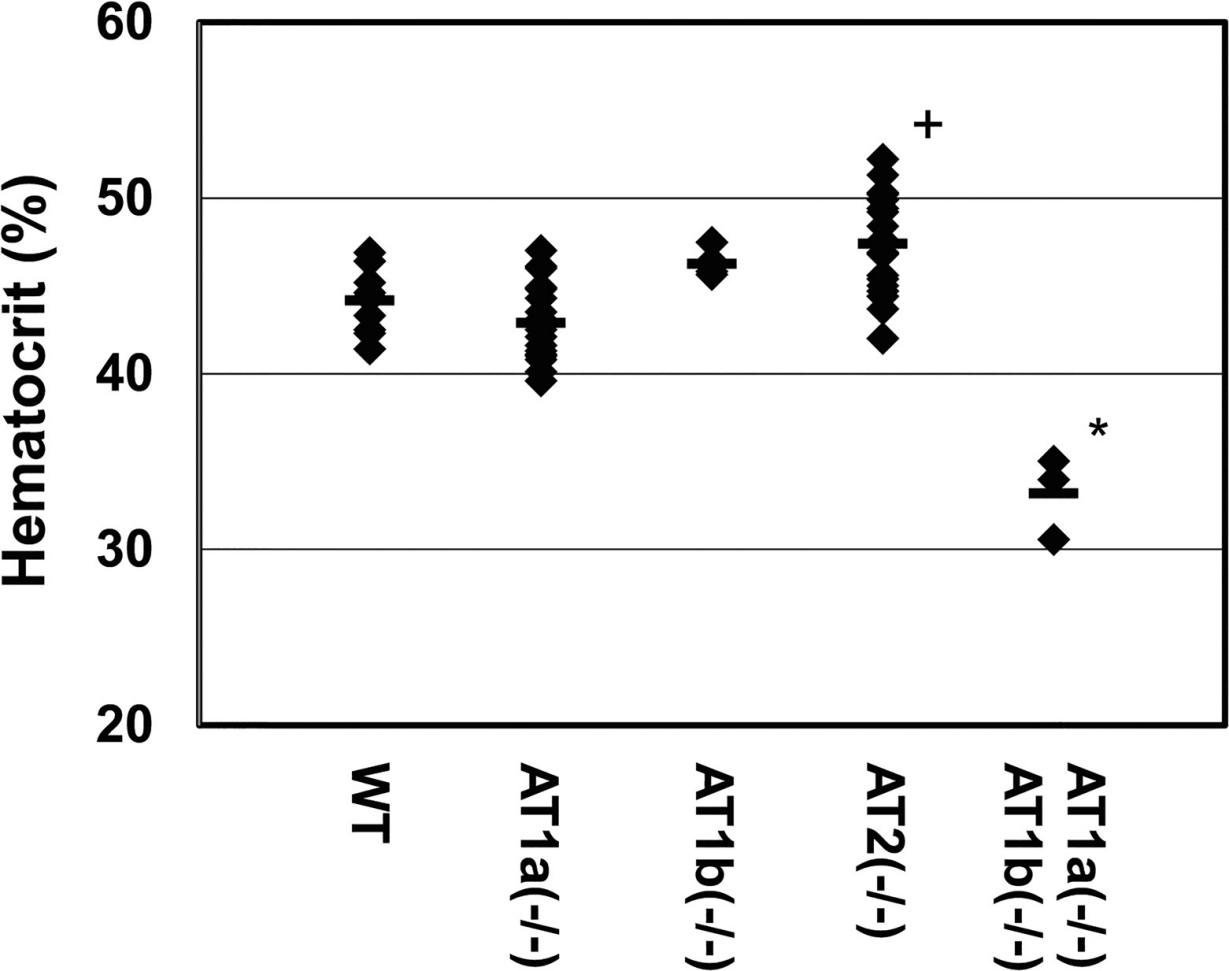
Hematocrit levels of Ang II receptor knockout mice. Hematocrit levels of AT1a(-/-) (n = 18), AT1a(+/-) AT1b(-/-) (n = 6), AT2(-/-) (n = 18), and AT1a(-/-) AT1b(-/-) (n = 3) mice were measured. The group mean is represented by a horizontal bar. (^+^ *p* < 0.05, *p < 0.001 vs. WT, ANOVA followed by Tukey’s HSD post hoc comparisons)

We next asked whether Ang II receptor knockout mice exhibit anemia. To this end, we examined hematocrit levels in animals deficient for the AT1a, AT1b, or AT2 receptor. None of these animals exhibited anemia: the hematocrit value of AT1a(-/-) was only slightly reduced relative to the WT [AT1a(-/-), 42.88 ± 2.20% (n = 18); *p* = 0.094 vs. WT; not significant]. Neither AT1b nor AT2 receptor–deficient mice exhibit anemia [AT1a(+/-) AT1b(-/-), 46.23 ± 0.68% (n = 6); not significant vs. WT; AT2(-/-), 47.39 ± 2.89% (n = 18); *p* = 0.0028 (ANOVA followed by Tukey’s HSD post hoc comparisons) vs. WT] (Fig 4). Curiously, the hematocrit level of AT2(-/-) was slightly elevated relative to WT (Fig 4).

### Genetic, but not pharmacological, inhibition of AT1 receptors reproduces anemia

Although anemia in Agt(-/-) was rescued via the Ang II–AT1 receptor pathway (Fig 3B), AT1a(-/-) exhibited almost normal erythropoiesis, whereas it exhibited severe hypotension close to that seen in the Agt(-/-) [4, 35]. Furthermore, numerous clinical studies have shown that the use of ARB is not associated with the progression of anemia [24–30, 41]. In light of these circumstances, we decided to examine whether AT1 receptor inhibition indeed causes anemia by pharmacological and genetic means.

**Fig 5:**
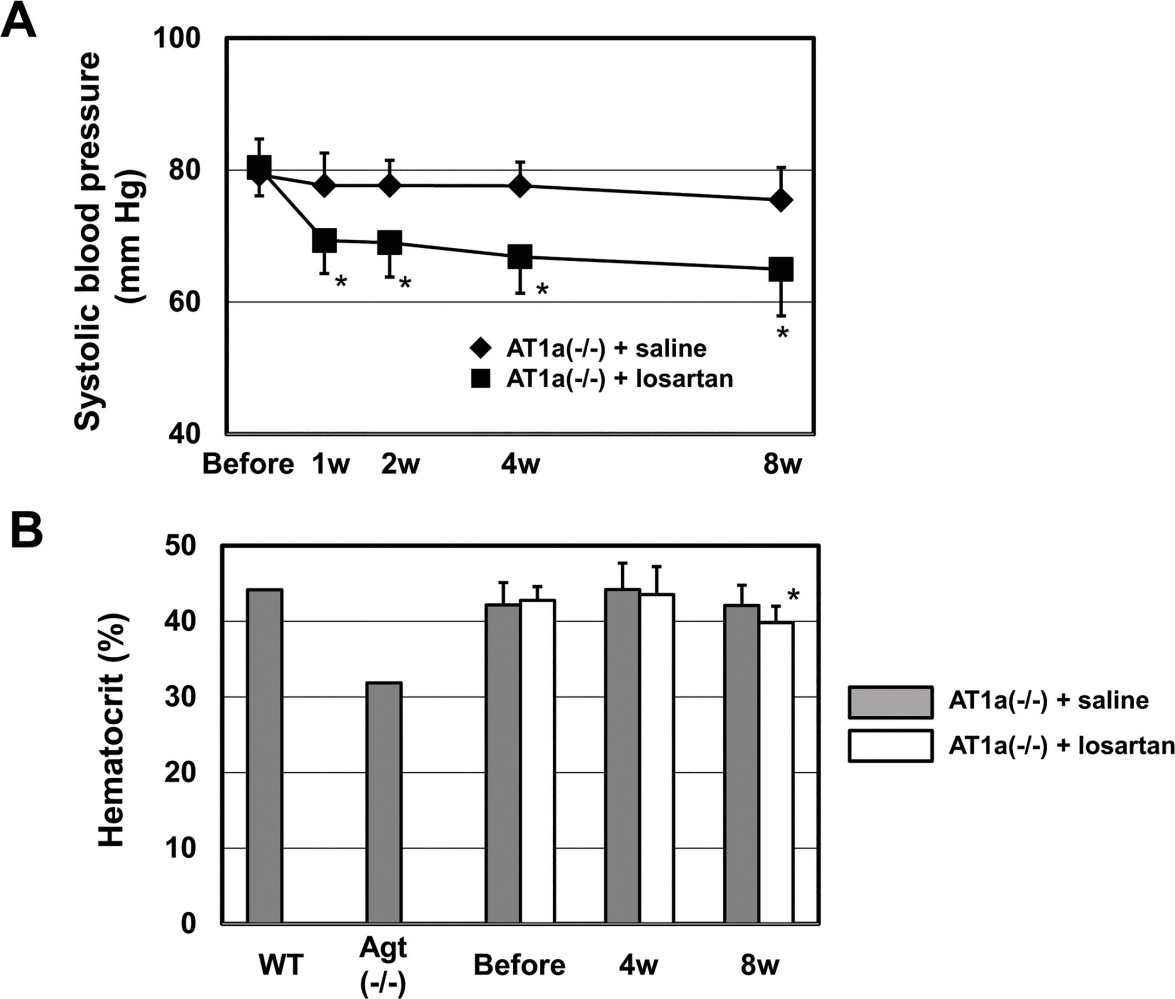
The systolic blood pressure and hematocrit values of AT1a(-/-) mice after oral administration of losartan. (A) Systolic blood pressures of AT1a(-/-) mice were measured before and after the oral administration of losartan (30 mg/kg/day). n = 7 for each group. (B) Hematocrit values of AT1a(-/-) mice were measured before and after oral administration of losartan. n = 10 for losartan and n = 11 for saline (*p < 0.05 vs. saline group). The hematocrit values of WT and Agt(-/-) are shown for comparison.

We hypothesized that the reason why AT1a(-/-) exhibited almost normal erythropoiesis was that the AT1b receptor plays compensatory roles in erythropoiesis in AT1a(-/-), although AT1b(-/-) itself exhibited no apparent phenotype [2]. To pharmacologically test this hypothesis, we orally administered AT1a(-/-) along with losartan to inhibit the remaining AT1b receptor in the animals. The AT1b receptor is pharmacologically indistinguishable from the AT1a receptor, and losartan efficiently inhibits both AT1a and AT1b receptors [42]. We measured blood pressure in addition to hematocrit values, before and after the administration of losartan, to confirm the effect of AT1b receptor blockade. As shown in Fig 5A, losartan significantly decreased the systolic blood pressure of AT1a(-/-) mice to levels comparable to the those in Agt(-/-) or AT1a(-/-) AT1b(-/-) mice; this effect was apparent from the first week of treatment, as reported previously [39]. However, losartan treatment only slightly decreased the hematocrit level in AT1a(-/-) mice, even after 8 weeks of administration, and it never reached the level in anemic Agt(-/-) mice [AT1a(-/-) + saline, 42.08 ± 2.68% (n = 11) and AT1a(-/-) + losartan, 39.83 ± 2.16% (n = 10), at 8 weeks; *p* < 0.05] (Fig 5B). Thus, pharmacological blockade of AT1b, in addition to genetic ablation of AT1a receptors, did not reproduce the anemic phenotype in the mice.

Therefore, to genetically test this hypothesis, we crossed AT1a(+/-) AT1b(-/-) and generated both AT1a- and AT1b-deficient mice. Double-homozygous mice deficient for both AT1a and AT1b receptor genes, AT1a(-/-) AT1b(-/-), exhibited significantly reduced hematocrit, comparable to that seen in Agt(-/-) animals [AT1a(-/-) AT1b(-/-), 33.18 ± 2.34% (n = 3); *p* < 0.001 (ANOVA followed by Tukey’s HSD post hoc comparisons) vs. WT] (Fig 4). These results clearly demonstrated that in regard to erythropoiesis, AT1a and AT1b can compensate for each other’s loss, and that while pharmacological inhibition of AT1 receptors cannot completely inhibit erythropoiesis, genetic ablation of AT1 receptors indeed causes anemia.

## Discussion

While the role of RAS in the blood pressure regulation has been widely appreciated, the impact of RAS inhibition on erythropoiesis was first suggested in the 1980s by the clinical observation that the use of ACEi was associated with progression of anemia in hypertensive patients [6–8]. Once ARBs became available, these anti-RAS drugs were found to cause a decrease in erythrocyte number in many clinical situations, such as essential hypertension [20], post-kidney transplant erythrocytosis [9, 19, 43], high-altitude polycythemia [10], hemodialysis and peritoneal dialysis [11, 16, 44], chronic renal failure [12, 45], heart failure [13], and cardiac surgery [14]. These hematocrit-reducing effects were also demonstrated in animal experiments by oral administration of ACEi or ARB to rat [46, 47]. By contrast, there have also been many studies suggesting that anti-RAS drugs do not inhibit erythropoiesis [24–30]. These contradictory results caused confusion in clinical fields regarding RAS inhibition and its possible effects on anemia.

Cole *et al*. previously reported that ACE knockout mice are anemic, with approximately 25% reduced hematocrit [40]. *In vitro*, ACE is capable of cleaving many small peptides besides angiotensin I, such as N-acetyl-Ser-Asp-Lys-Pro (acetyl-SDKP), bradykinin, Substance P, and luteinizing hormone–releasing hormone. Acetyl-SDKP has been implicated as a bone marrow stem-cell suppressor [48, 49]. They investigated the possible role of Ang II in progression of anemia of ACE knockout mice by administering a small dose of Ang II, observed recovery of hematocrit to near normal levels and speculated that the cause of anemia found in ACE knockout mice was likely to be a lack of Ang II, but not the accumulation of acetyl-SDKP [40]. However, our group and Billet et al. previously showed that Ang II itself has potent erythropoietic activity and stimulates Epo production via AT1 receptors [4, 5]. Therefore, the possibility still remains that exogenously administered Ang II somehow rescued the anemic phenotype of ACE knockout mice. Our finding that Agt(-/-) and Ren(-/-) that lack Ang II production displayed the same degree of anemic phenotype strongly suggests that the observed anemia was completely due to the absence of Ang II, rather than the absence of other substrates (Fig 1A). Since the two lines of knockout mice are normocytic and normochromic anemia (Table 1), and morphology of peripheral blood smears were normal, the anemia seems to be attributable to the reduction in mature red blood cell production, not to the disruption of red blood cells. In humans, the half-life of red blood cells ranges from 25 to 36 days [50], and many clinical studies have shown that hematocrit levels reach a nadir within three months after the use of RAS inhibitors, and then remain stable over a long period [21, 51, 52]. Considering the time-course of anemia progression, RAS-inhibitors seem to reduce red blood cell production in humans.

The mechanisms of anemia caused by AT1 receptor inhibition have remained unknown. Epo, the most essential hormonal regulator of erythropoiesis, is primarily produced in the kidney and liver. Under physiological conditions, plasma Epo levels are inversely correlated with the O_2_-delivering capacity of red blood cells [53]. Kim et al. revealed Ang II–induced Epo expression *in situ* in murine kidney slices and 786-O kidney cells; they also found that Epo expression is mediated via the AT1 receptor, which acts upstream of the ERK and Egr-1 pathways, *in vitro* [54]. Even though there remain several mechanisms of Ang II–induced Epo production, such as hemodynamic effects and tissue oxygenation of Epo-producing cells, these *in vitro* results indicate the possible direct effects of Ang II on Epo-producing cells.

In contrast, *in vivo* relationship between Ang II-AT1 inhibition and Epo levels is still controversial. In human studies, treatment by ACE inhibitors or ARBs has been associated with variable, decreased, or unchanged levels of plasma erythropoietin [32]. In animal models, ACE knockout mice have elevated Epo levels relative to WT [40], while other report showed the decrease of Epo levels after the use of ACE inhibitors or ARBs [47, 55]. We examined anemic Agt(-/-) mice and found slightly elevated, but not significantly different plasma Epo levels relative to the WT (Fig 2A). Furthermore, Agt(-/-) mice had the potential to further increase plasma Epo when they were subjected to bleeding (Fig 2A and 2B). Thus, Epo production does not seem to be impaired in Agt(-/-) mice. As a recent study demonstrated the paracrine production of Epo in the liver, especially in anemic condition [56], the extra-renal compensatory production mediated by complex hypoxia-inducible factors (HIFs) in Epo producing cells [54, 55] might explain the discrepancy between *in vitro* and *in vivo* results.

To date, several other possible explanations for anemia have been proposed, such as direct apoptotic effects of ACEi on erythroid precursor cells [57, 58], drug toxicity [46, 47], and accumulation of substrates of renin or ACE. However, based on our results obtained from genetically engineered mice, these proposed mechanisms do not seem likely; instead, the Ang II-AT1 receptor pathway appears to be crucial. Among AT1 receptor subtypes present in mammals, AT1a receptor is considered to be predominant, at least in blood pressure regulation [35]. AT1b receptor expression is detected only in the limited organs, such as brain, testis, and adrenal gland, and is absent or faint in the kidney, liver, and bone marrow cells [59]. The organs in which AT1a and AT1b receptors mutually compensate for erythropoiesis still remain to be elucidated.

**Fig 6:**
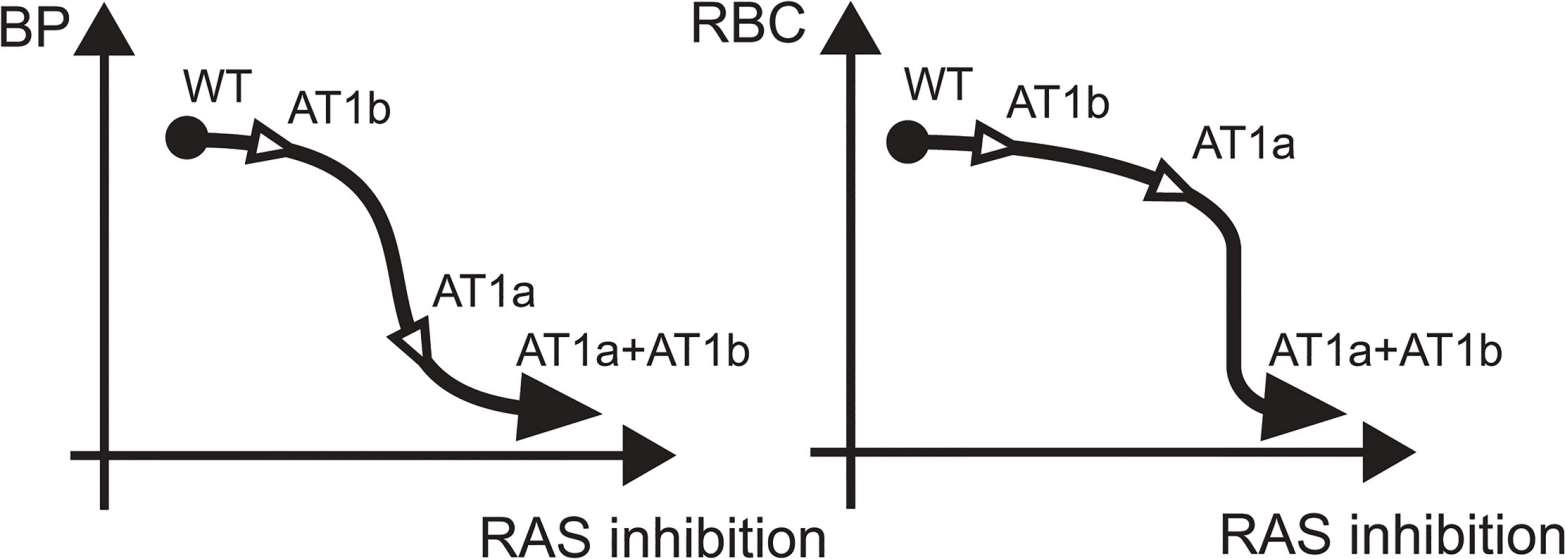
Schematic view of the correlation between AT1 receptor inhibition (horizontal) and blood pressure (BP) or erythropoiesis (RBC) (vertical).

Based on our data and previous findings [2, 4, 33–36, 40], we propose a hypothetical scheme that graphically depicts the correlation between intensity of AT1 receptor inhibition and blood pressure or erythropoiesis (Fig 6). In designing this scheme, we took the following previous studies and present data into consideration; i) AT1a(-/-) mice exhibit hypotension [80 mmHg in AT1a(-/-) vs. 110 mmHg in WT] [35] but not anemia (Fig 4); ii) AT1b(-/-) exhibits normal blood pressure [2] and normal erythropoiesis (Fig 4); iii) Agt(-/-), Ren(-/-), ACE knockout, and AT1a(-/-) AT1b(-/-) mice exhibited almost the same degree of hypotension (70 mmHg in these mice vs. 110 mmHg in WT) [33, 34, 36, 40] and anemia (Fig 4); and iv) the hematocrit value was more resistant than blood pressure to pharmacological inhibition of AT1 receptor (Fig 5). Our results provide novel insights into the clinical physiology of RAS and erythropoiesis.
